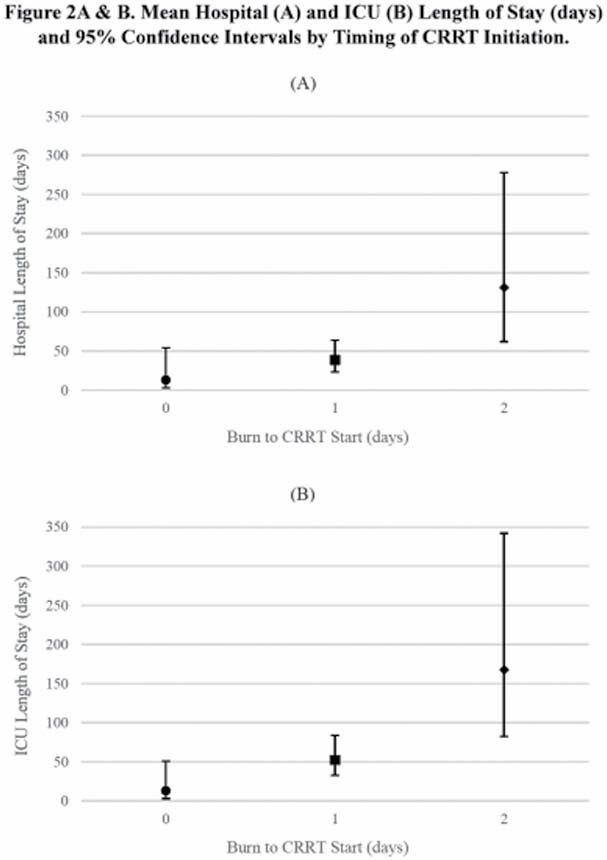# 3 Continuous Renal Replacement Therapy for the Treatment of Burn Shock: A Post Hoc Analysis

**DOI:** 10.1093/jbcr/irac012.007

**Published:** 2022-03-23

**Authors:** Anthony P Basel, Garrett W Britton, Jordan Evans, David Dado, Laura L Scott, Lonnie E Grantham, Leopoldo C Cancio, Kevin K Chung

**Affiliations:** United States Army Institute of Surgical Research, Fort Sam Houston, Texas; US Army Institute of Surgical Research, JBSA Fort Sam Houston, Texas; Brooke Army Medical Center, Fort Sam Houston, Texas; Brooke Army Medical Center, Fort Sam Houston, Texas; United States Army Institute of Surgical Research, Fort Sam Houston, Texas; United States Army Institute of Surgical Research, Fort Sam Houston, Texas; US Army Institute of Surgical Research, JBSA Fort Sam Houston, Texas; Uniformed Services University, Bethesda, Maryland

## Abstract

**Introduction:**

Burn shock is a consequence of burns that cover ≥20% TBSA and may be complicated by acute kidney injury, which is commonly treated with continuous renal replacement therapy (CRRT). However, early initiation of CRRT has not been clinically evaluated for the treatment of burn shock.

**Methods:**

Data were obtained from the *Renal Replacement Therapy in Severe Burns: A Multicenter Observational Study*. In that study, baseline (t_0_) measurements were taken at the time of CRRT initiation and ~24 (t_1_) and ~48 (t_2_) hours thereafter. Patients were included in this analysis if they had ≥20% TBSA and began CRRT within 2 days of injury. Patients were categorized as Group A (began CRRT on same day as injury), Group B (began CRRT on day 1 postburn), and Group C (began CRRT on day 2 postburn). Outcomes measured at t_0_, t_1_, and t_2_ and hospital and ICU length of stay (LOS) were analyzed using generalized linear mixed models. Cox proportional hazards models were used to assess survival to hospital discharge (HD). All models were adjusted, e.g. for age, % full thickness, etc. Burn center was included as a random effect.

**Results:**

More than half of the 48 patients included were treated at just 2 burn centers. Timing of CRRT initiation varied by center, with all patients at one center starting CRRT on either the day of injury or the day after injury. Nearly 96% of patients had AKI at CRRT start and, of those, 22 were at stage 1 or 2. Patients generally had severe burns; Group A had more inhalation injuries and higher %TBSA, % full thickness, and Baux scores than Groups B and C. Shock index (SI) was persistently elevated across all 3 time points and did not vary by timing of CRRT initiation (p=0.37). Vasopressor dependency index (VDI) was also not associated with timing of CRRT initiation (p >0.99), although mean VDI for Groups B and C declined over time. For all 3 groups, fluid balance decreased from t_0_, but there were no differences among the groups (all p >0.30). Survival to HD was better for patients with lower TBSA (i.e. 20-49%) compared to those with TBSA ≥50% (hazard ratio=0.37; 95% CI=0.15-0.91). In contrast, timing of CRRT initiation was not associated with survival (p=0.73). Among patients that survived to HD, the mean hospital LOS was shorter for Groups A (13 days; p=0.01) and B (39 days; p=0.03) compared to Group C (131 days). Mean ICU LOS was also shorter for Groups A (13 days; p=0.01) and B (52 days; p=0.03) than for Group C (168 days).

**Conclusions:**

In this analysis, earlier initiation of CRRT did not improve survival to hospital discharge. Nonetheless, starting patients on CRRT early may be advantageous for reducing ICU and hospital LOS for those patients that do survive.